# *Usp14* deficiency removes α-synuclein by regulating S100A8/A9 in Parkinson’s disease

**DOI:** 10.1007/s00018-024-05246-8

**Published:** 2024-05-23

**Authors:** Liuyan Ding, Lin Lu, Shaohui Zheng, Zhiling Zhang, Xingting Huang, Runfang Ma, Mengran Zhang, Zongtang Xu, Minshan Chen, Zhimei Guo, Si Zhu, Junwei Gong, Hengxu Mao, Wenlong Zhang, Pingyi Xu

**Affiliations:** 1 Department of Neurology, The First Affiliated Hospital of Guangzhou Medical University, Guangzhou, China; 2 Key Laboratory of Neurological Function and Health, School of Basic Medical Sciences, Guangzhou Medical University, Guangzhou, China; 3 School of Life Sciences, Westlake University, Hangzhou, China; 4 Westlake Laboratory of Life Sciences and Biomedicine, Hangzhou, China

**Keywords:** Parkinson’s disease, USP14, α-synuclein, S100A8/A9, Autophagy

## Abstract

Ubiquitin-proteasome system dysfunction triggers α-synuclein aggregation, a hallmark of neurodegenerative diseases, such as Parkinson’s disease (PD). However, the crosstalk between deubiquitinating enzyme (DUBs) and α-synuclein pathology remains unclear. In this study, we observed a decrease in the level of ubiquitin-specific protease 14 (USP14), a DUB, in the cerebrospinal fluid (CSF) of PD patients, particularly females. Moreover, CSF USP14 exhibited a dual correlation with α-synuclein in male and female PD patients. To investigate the impact of USP14 deficiency, we crossed USP14 heterozygous mouse (USP14^+/−^) with transgenic A53T PD mouse (A53T-Tg) or injected adeno-associated virus (AAV) carrying human α-synuclein (AAV-*h*α-Syn) in USP14^+/−^ mice. We found that *Usp14* deficiency improved the behavioral abnormities and pathological α-synuclein deposition in female A53T-Tg or AAV-*h*α-Syn mice. Additionally, *Usp14* inactivation attenuates the pro-inflammatory response in female AAV-*h*α-Syn mice, whereas *Usp14* inactivation demonstrated opposite effects in male AAV-*h*α-Syn mice. Mechanistically, the heterodimeric protein S100A8/A9 may be the downstream target of *Usp14* deficiency in female mouse models of α-synucleinopathies. Furthermore, upregulated S100A8/A9 was responsible for α-synuclein degradation by autophagy and the suppression of the pro-inflammatory response in microglia after *Usp14* knockdown. Consequently, our study suggests that USP14 could serve as a novel therapeutic target in PD.

**Supplementary Information:**

The online version contains supplementary material available at 10.1007/s00018-024-05246-8.

## Introduction

Parkinson’s disease (PD) is a progressive neurodegenerative disorder characterized by impaired movements, including tremor and bradykinesia [1]. Pathologically, PD is marked by the loss of dopaminergic neurons and the presence of Lewy body inclusions composed of aggregated α-synuclein in the nigrostriatal pathway [2, 3]. Additionally, *SNCA* (encoding α-synuclein) mutations are critical genetic risk factors for PD [4–6], emphasizing the significance of α-synuclein pathology in PD pathogenesis. The ubiquitin proteasome system degrades misfolded proteins, and its malfunction contributes to the formation of α-synuclein aggregates in PD [7]. In the classical ubiquitin proteasome system pathway, ubiquitin-activating enzyme (E1) is recruited to activate ubiquitin, which is then transferred to the ubiquitin-conjugating enzyme (E2) and subsequently conjugated to lysine residues within the substrate by ubiquitin ligase (E3). Several lysine residues, such as K12, K21, K23, and K34, have been identified in α-synuclein [8, 9]. Deubiquitinating enzymes (DUBs) can cleave ubiquitin from proteins, stabilizing the substrate. A deubiquitination protein, ubiquitin C-terminal hydrolase L1 (*UCHL1*), is considered a risk gene for PD [10, 11]. It loss stabilizes pyruvate kinase and induces energy-dependent mitophagy, improving PD-related phenotypes [12]. Furthermore, S-nitrosylation of UCHL1 promotes α-synuclein aggregation [13], suggesting its close relationship with PD pathology. However, it is unclear how other DUBs contribute to the pathogenesis of PD.

Ubiquitin-specific protease 14 (USP14), a key DUB, is associated with proteasomes and plays an important role in protein degradation in multiple diseases, such as cancer, inflammatory diseases, and neurodegenerative disorders [14–19]. In the brain, USP14 is crucial for neuronal survival, mitophagy, neuroinflammation, and neuromuscular junction structure and function [20–25]. USP14 inhibition activates the proteasome, removes tau oligomers, and promotes huntingtin aggregation [19], suggesting that it contributes to aggregate deposition in neurodegenerative diseases. However, it remains unclear whether USP14 regulates α-synuclein pathology.

In the present study, we found reduced USP14 level in the cerebrospinal fluid (CSF) of PD patients, particularly females. Intriguingly, CSF USP14 was positively correlated with α-synuclein in male PD patients but negatively correlated in female PD patients. We crossed USP14 heterozygous mice (USP14^+/−^) with transgenic A53T PD mice (A53T-Tg) and injected adeno-associated virus (AAV) carrying human α-synuclein (AAV-*h*α-Syn) in USP14^+/−^ mice. *Usp14* deficiency attenuated the behavioral abnormalities and pathological α-synuclein deposition in female A53T-Tg or AAV-*h*α-Syn mice. Furthermore, *Usp14* inactivation attenuates the pro-inflammatory response in female AAV-*h*α-Syn mice. Mechanistically, *Usp14* deficiency enhanced the expressions of heterodimeric protein S100A8/A9 in the substantia nigra (SN) of mouse models with α-synucleinopathies. Increased S100A8/A9 degrades α-synuclein by autophagy and suppresses the pro-inflammatory response to *Usp14* knockdown. Collectively, our study indicates that USP14 is a novel regulator of α-synuclein in PD.

## Results

### USP14 is correlated with α-synuclein level in PD patients

**Fig. 1:**
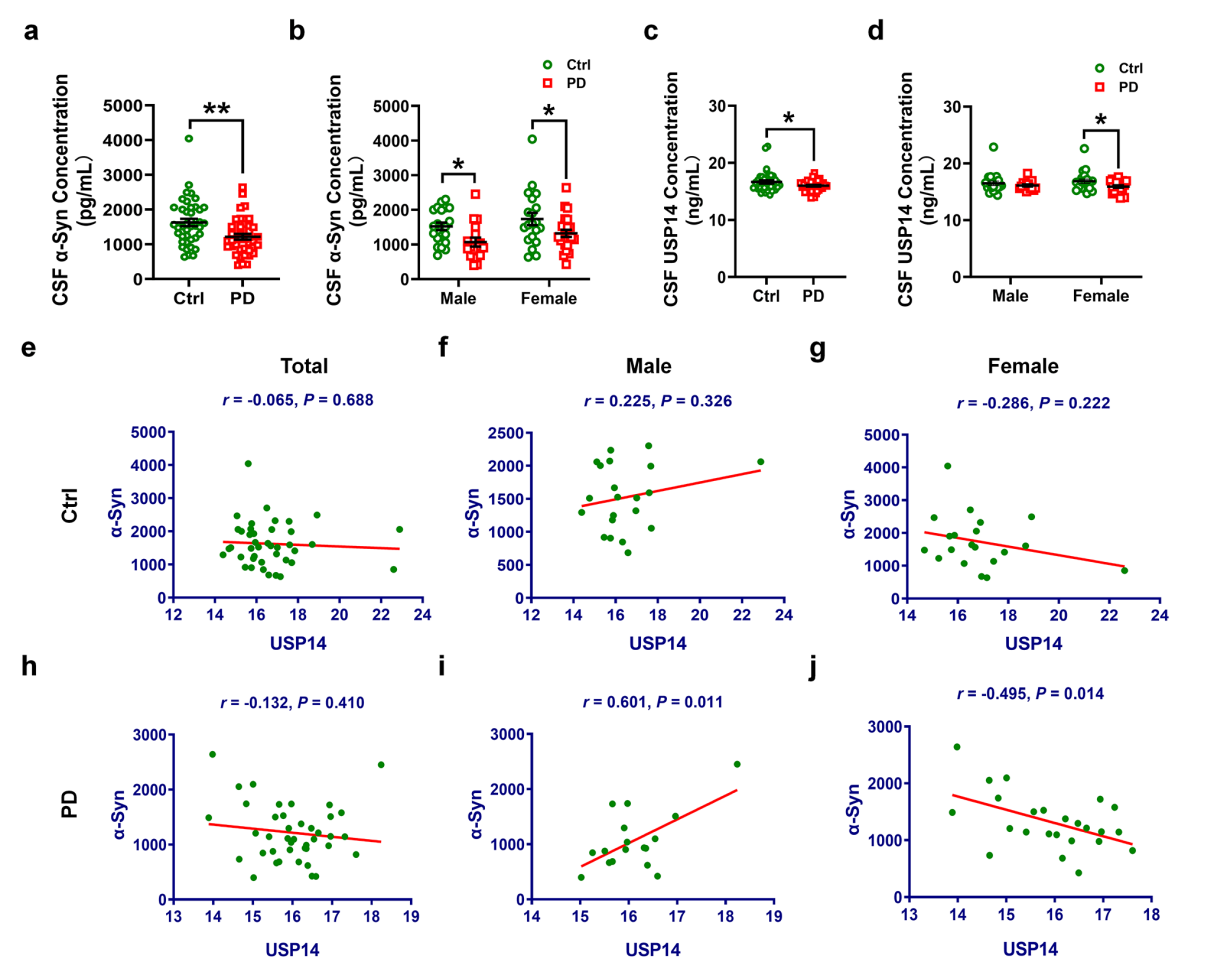
USP14 demonstrates dual correlation with α-synuclein in the CSF of PD patients. (**a–d**) The CSF levels of α-synuclein and USP14 in male and female controls (*n* = 41) and PD patients (*n* = 41) were assessed using ELISA. The control group included 21 males and 20 females, whereas the PD group included 17 males and 24 females. (**e–j**) Correlation between USP14 and α-synuclein in control and PD groups was assessed by Pearson’s correlation analysis. Results are expressed as mean ± SEM. ^**^p < 0.01, ^*^p < 0.05 vs. controls. Statistical significance was determined using Student’s *t*-test

Since USP14 inhibition promotes PINK1/Parkin-mediated mitophagy [26], and USP14 modulates the degradation of tyrosine hydroxylase [27], the rate-limiting enzyme in dopamine biosynthesis, we hypothesize that USP14 may be related to PD. First, we detected the CSF levels of α-synuclein and USP14 in controls and PD patients. Consistent with a previous study [28], CSF α-synuclein level was significantly decreased in both male and female PD patients (Fig. 1a and b). In the present study, the CSF USP14 level was lower in PD patients, particularly females (Fig. 1c and d). Pearson correlation analysis demonstrated no correlation between USP14 and α-synuclein levels in the control group (Fig. 1e–g). However, CSF USP14 level was positively related to the α-synuclein level in male PD patients and negatively related in female PD patients (Fig. 1h–j). These data suggest that the relationship between USP14 level and α-synuclein pathology differs between genders.

### *Usp14* deficiency improves motor deficits and reduces pathological α-synuclein deposition in female A53T PD mice

**Fig. 2:**
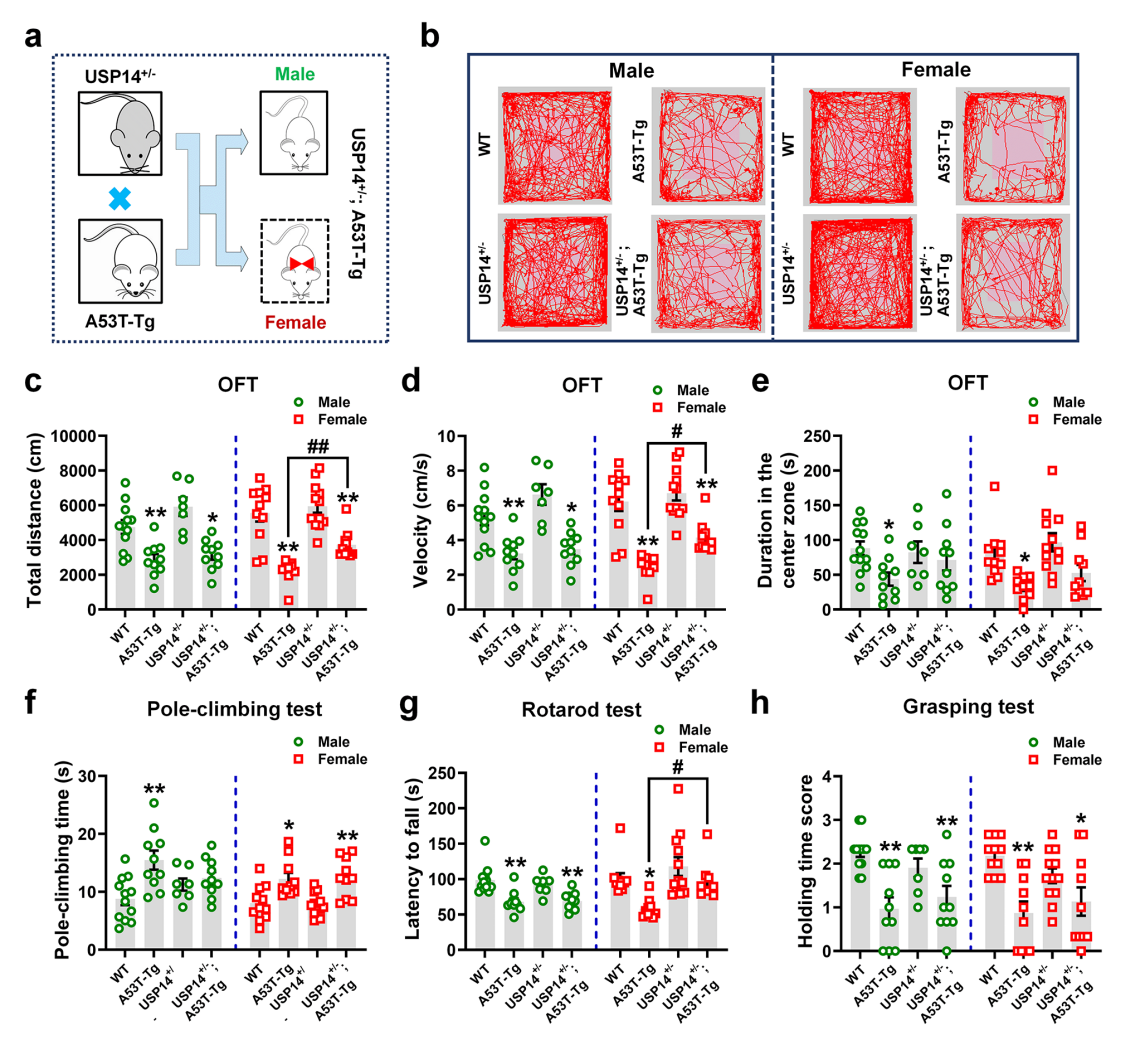
*Usp14* deficiency ameliorates the motor deficits in female A53T-Tg mice. (**a**) Crossing of USP14^+/−^ and A53T-Tg mice. (**b–e**) Representative traces, total travelled distance, movement speed, and time spent in the center zone in the open-field test. (**f**) Pole climbing test was used to examine bradykinesia in mice. (**g**) Rotarod test was used to examine motor coordination in mice. (**h**) Grasping test was used to examine the grip strength in mice. *n* = 12, 10, 7, and 10 for male WT, A53T-Tg, USP14^+/−^, and USP14^+/−^; A53T-Tg mice, respectively; *n* = 11, 10, 12, and 10 for female WT, A53T-Tg, USP14^+/−^, and USP14^+/−^; A53T-Tg mice, respectively. Results are expressed as mean ± SEM. ^**^p < 0.01, ^*^p < 0.05 vs. WT mice; ^##^*p* < 0.01, ^#^*p* < 0.05 vs. A53T-Tg mice. Statistical significance was determined using one-way ANOVA and Tukey’s test for *post hoc* comparisons

We crossed USP14 heterozygous mouse (USP14^+/−^) with transgenic A53T PD mouse (A53T-Tg) to generate male and female USP14^+/−^; A53T-Tg mice (Fig. 2a). Open-field test (OFT) demonstrated reduced travelled distance, movement speed, and time spent in the center zone in male and female A53T-Tg mice (Fig. 2b–e). However, female USP14^+/−^; A53T-Tg mice exhibited increased travelled distance and movement speed compared to A53T-Tg mice (Fig. 2b–e). Furthermore, A53T-Tg mice exhibited motor impairments, as evidenced by prolonged climbing time, worsening motor coordination, and weakening grip strength (Fig. 2f–h). Female USP14^+/−^; A53T-Tg mice demonstrated enhanced motor activity and grip strength in the rotarod and grasping tests, respectively, compared to A53T-Tg mice (Fig. 2g and h). These data suggest that *Usp14* deficiency ameliorates motor deficits in female A53T-Tg mice.

**Fig. 3:**
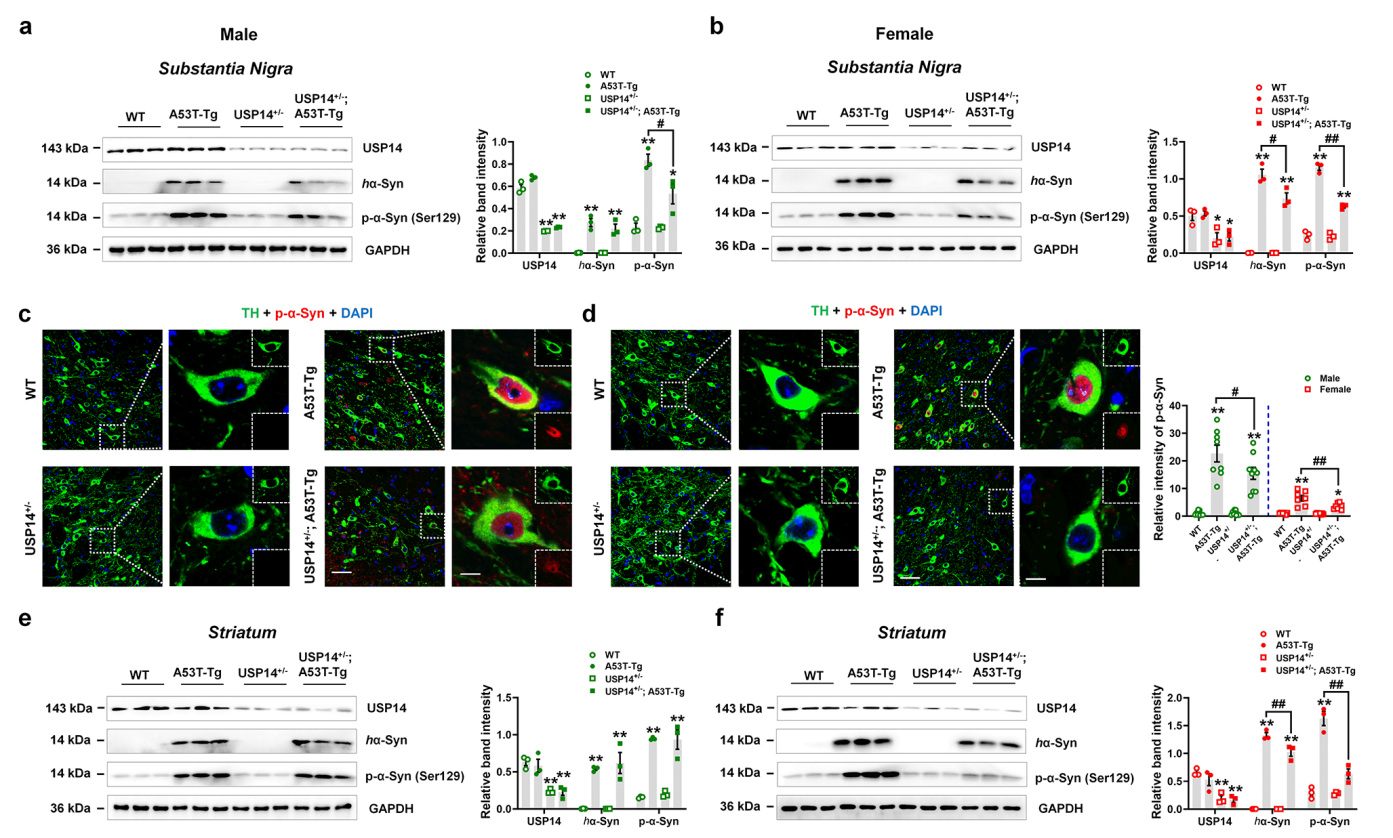
*Usp14* deficiency attenuates α-synuclein deposition in female A53T-Tg mice. (**a** and **b**) Representative blots and quantification showing the expressions of USP14, human α-synuclein (*h*α-Syn; α-synuclein was abbreviated as α-Syn in the Figure legends), and p-α-Syn (Ser129) in the SN of male and female WT, A53T-Tg, USP14^+/−^, and USP14^+/−^; A53T-Tg mice. *n* = 3 per group. (**c** and **d**) Immunostaining and quantification of the intensity of *p-α-Syn* in TH-positive cells in the SNpc of male and female WT, A53T-Tg, USP14^+/−^, and USP14^+/−^; A53T-Tg mice. *n* = 9, 8, 9, and 9 for male WT, A53T-Tg, USP14^+/−^, and USP14^+/−^; A53T-Tg mice, respectively; *n* = 6, 7, 7, and 8 for female WT, A53T-Tg, USP14^+/−^, and USP14^+/−^; A53T-Tg mice, respectively. Scale bar, 50 μm. Magnified images are shown on the right side. Scale bar, 7 μm. (**e** and **f**) Representative blots and quantification of the expressions of USP14, *h*α-Syn, and p-α-Syn (Ser129) in the striatum of male and female WT, A53T-Tg, USP14^+/−^, and USP14^+/−^; A53T-Tg mice. *n* = 3 per group. Results are expressed as mean ± SEM. ^**^p < 0.01, ^*^p < 0.05 vs. WT mice; ^##^*p* < 0.01, ^#^*p* < 0.05 vs. A53T-Tg mice. Statistical significance was determined using one-way ANOVA and Tukey’s test for *post hoc* comparisons

We assessed α-synuclein deposition under *Usp14* deficiency. Exogenous human α-synuclein and endogenous phosphorylated α-synuclein at Ser129, a key hallmark of Lewy bodies, were significantly increased in the SN and striatum of male and female A53T-Tg mice (Fig. 3a–f). USP14^+/−^; A53T-Tg mice showed reduced expression of phosphorylated α-synuclein compared to male and female A53T-Tg mice, whereas female mice had reduced human α-synuclein expression in the SN (Fig. 3a–d). Moreover, compared to A53T-Tg mice, the expressions of human α-synuclein and phosphorylated α-synuclein were inhibited in the striatum of female USP14^+/−^; A53T-Tg mice (Fig. 3e and f). These results suggest that *Usp14* deficiency reversed α-synuclein deposition in female A53T-Tg mice.

### *Usp14* inactivation increased S100A8/A9 expression in female A53T-Tg mice

**Fig. 4:**
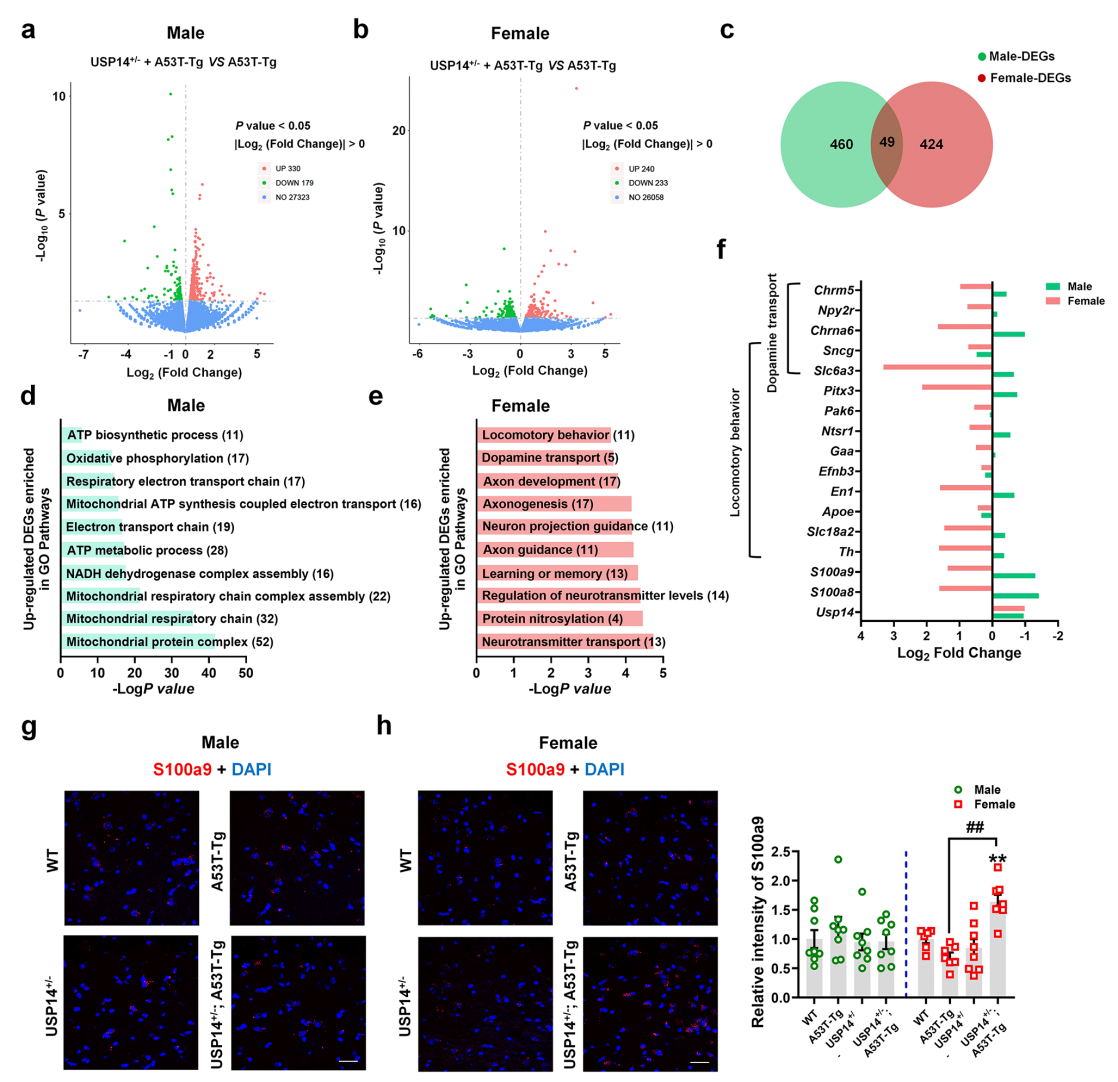
*Usp14* inactivation increased S100A8/A9 expression in female A53T-Tg mice. (**a** and **b**) Volcano plot showing the DEGs between male and female USP14^+/−^; A53T-Tg and A53T-Tg mice. (**c**) Venn diagram showing the overlapped genes between male and female USP14^+/−^; A53T-Tg vs. A53T-Tg DEGs. (**d**) Gene ontology (GO) pathways enriched by the upregulated DEGs between male USP14^+/−^; A53T-Tg and A53T-Tg mice. (**e**) GO pathways enriched by the upregulated DEGs between female USP14^+/−^; A53T-Tg and A53T-Tg mice. (**f**) Representative expressions of DEGs, including *Usp14*, *S100A8*, and *S100A9*, and the DEGs enriched in the locomotor behavior and dopamine transport pathways in male and female mice. (**g** and **h**) Immunostaining and quantification of the S100a9 intensity in the SNpc of male and female WT, A53T-Tg, USP14^+/−^, and USP14^+/−^; A53T-Tg mice. *n* = 8 per group for male mice; *n* = 6, 7, 8, and 8 for female WT, A53T-Tg, USP14^+/−^, and USP14^+/−^; A53T-Tg mice, respectively. Scale bar, 30 μm. Results are expressed as mean ± SEM. ^**^p < 0.01 vs. WT; ^##^*p* < 0.01 vs. A53T-Tg. Statistical significance was determined using one-way ANOVA and Tukey’s test for *post hoc* comparisons

We used RNA-sequencing (RNA-seq) to explore the mechanisms underlying the aforementioned changes. We explored the differentially expressed genes (DEGs) in the SN between male and female A53T-Tg and USP14^+/−^; A53T-Tg mice (Fig. 4a and b; Supplementary Fig. 1a and b). In total, 49 DEGs were shared by male and female mice (Fig. 4c). In male mice, gene ontology pathways enriched by the upregulated DEGs were related to mitochondrial function, such as the mitochondrial respiratory chain and ATP metabolism (Fig. 4d), whereas in female mice, the pathways were related to synaptic function, such as neurotransmitter transport, axon development, and dopamine transport (Fig. 4e). We investigated the DEGs enriched in locomotor behavior and dopamine transport, which were closely related to PD (Fig. 4f). Notably, the expression of most DEGs enriched in these pathways was increased in female mice and decreased in male mice (Fig. 4f). Furthermore, the expression patterns of *S100A8* and *S100A9* demonstrated a similar pattern (Fig. 4f), with the S100A8/A9 expression being increased in the SN pars compacta (SNpc) of USP14^+/−^; A53T-Tg mice compared to A53T-Tg mice (Fig. 4g and h).

### *Usp14* suppression showed opposite effects on motor function and α-synuclein deposition in male and female AAV-*h*α-Syn mice

**Fig. 5:**
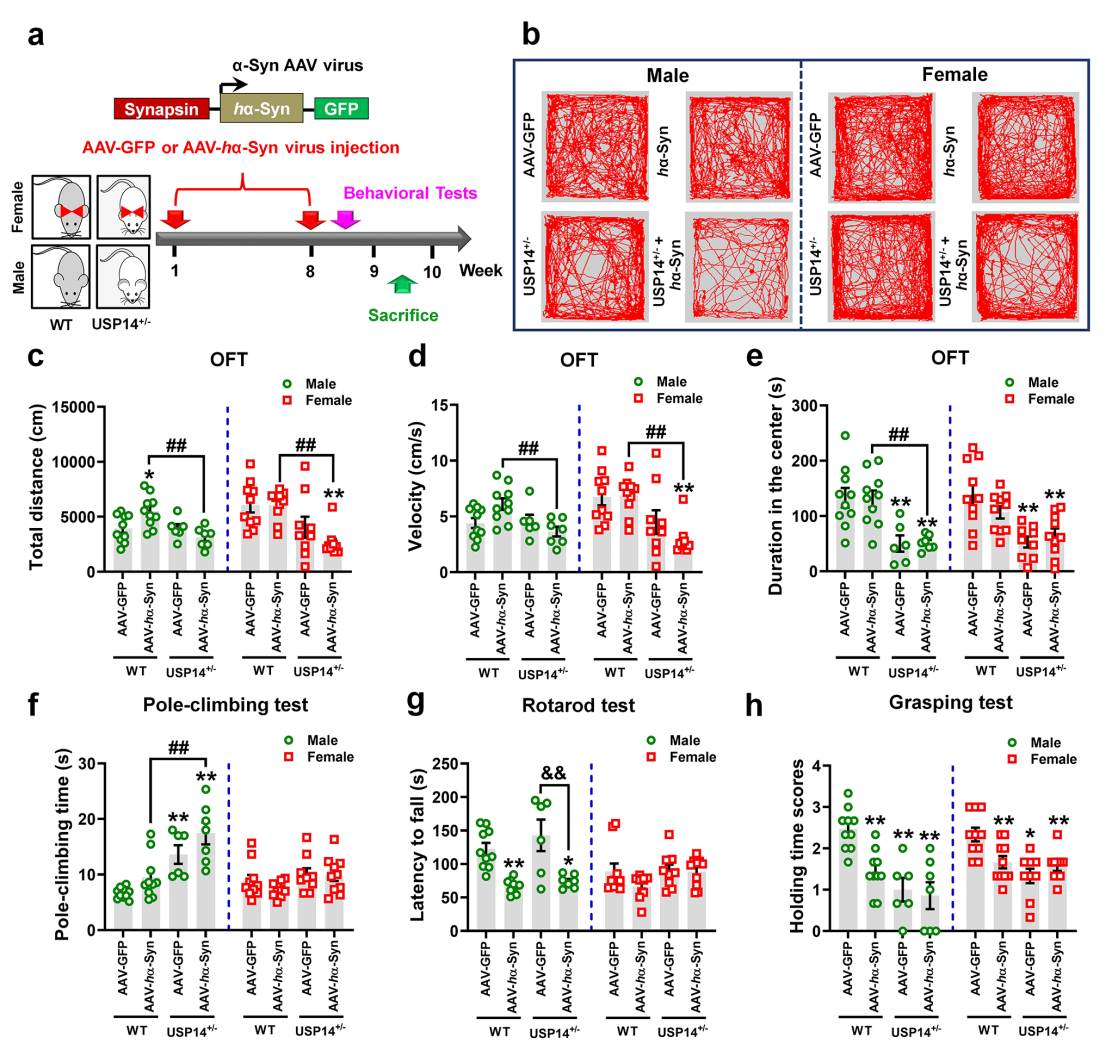
*Usp14* deficiency improves the motor deficits in female AAV-*h* α-Syn-injected mice. (**a**) Experimental design for AAV-*h*α-Syn virus administration in male and female WT and USP14^+/−^ mice. (**b**–**e**) Representative traces, total travelled distance, movement speed, and time spent in the center zone in the open-field test. (**f**) Pole climbing test was used to examine bradykinesia in mice. (**g**) Rotarod test was used to examine the motor coordination in mice. (**h**) Grasping test was used to examine the grip strength in mice. *n* = 10, 10, 6, and 7 for male AAV-GFP, AAV-*h*α-Syn, USP14^+/−^ + AAV-GFP, and USP14^+/−^ + AAV-*h*α-Syn mice, respectively; *n* = 10, 10, 9, and 10 for female AAV-GFP, AAV-*h*α-Syn, USP14^+/−^ + AAV-GFP, and USP14^+/−^ + AAV-*h*α-Syn mice, respectively. Results are expressed as mean ± SEM. ^**^p < 0.01, ^*^p < 0.05 vs. AAV-GFP; ^##^*p* < 0.01, ^#^*p* < 0.05 vs. AAV-*h*α-Syn. Statistical significance was determined using one-way ANOVA and Tukey’s test for *post hoc* comparisons

In addition to crossing USP14^+/−^ and A53T-Tg mice, we injected AAV-*h*α-Syn in the SNpc of male and female USP14^+/−^ mice (Fig. 5a). Compared to AAV-*h*α-Syn, male USP14^+/−^ + AAV-*h*α-Syn mice demonstrated reduced travelled distance, movement speed, and the time spent in the center zone in OFT (Fig. 5b–e). Furthermore, male USP14^+/−^ + AAV-*h*α-Syn mice exhibited a longer climbing time compared to AAV-*h*α-Syn mice (Fig. 5f). These results suggest *Usp14* inactivation damaged the motor function following AAV-*h*α-Syn administration. Female USP14^+/−^ + AAV-*h*α-Syn mice demonstrated improved motor coordination compared to AAV-*h*α-Syn mice (Fig. 5g), indicating *Usp14* inactivation may be beneficial for AAV-*h*α-Syn administration. The grip strength was not significantly different between male and female AAV-*h*α-Syn and USP14^+/−^ + AAV-*h*α-Syn mice (Fig. 5h).

**Fig. 6:**
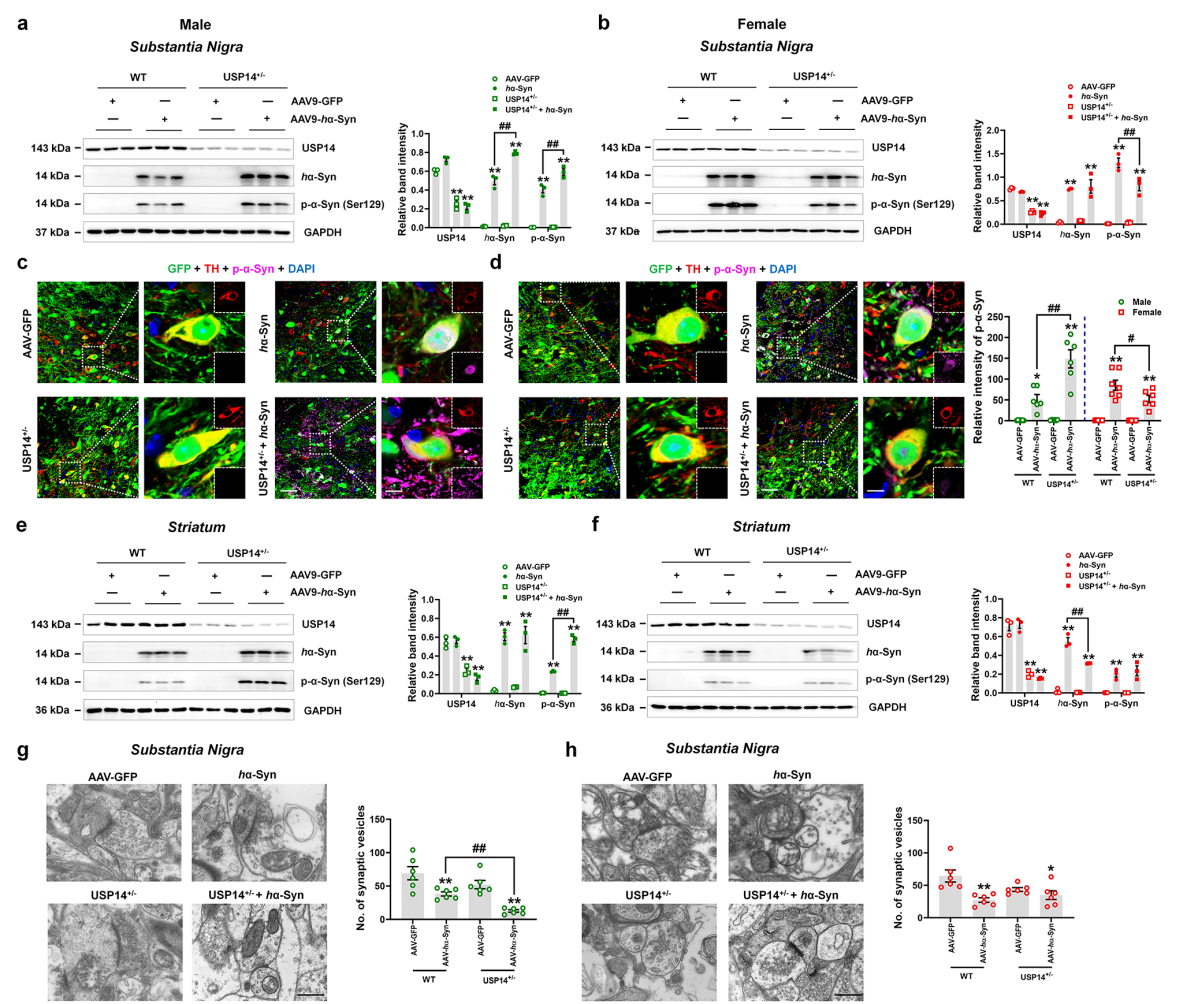
*Usp14* deficiency demonstrates opposite effects on α-synuclein deposition in male and female AAV-*h*α-Syn-injected mice. (**a** and **b**) Representative blots and quantification showing the expressions of USP14, *h*α-Syn, and p-α-Syn (Ser129) in the SN of male and female AAV-GFP, AAV-*h*α-Syn, USP14^+/−^ + AAV-GFP, and USP14^+/−^ + AAV-*h*α-Syn mice. *n* = 3 per group. (**c** and **d**) Immunostaining and quantification of the p-α-Syn intensity in TH-positive cells in the SNpc of male and female AAV-GFP, AAV-*h*α-Syn, USP14^+/−^ + AAV-GFP, and USP14^+/−^ + AAV-*h*α-Syn mice. *n* = 7, 6, 6, and 6 for male AAV-GFP, AAV-*h*α-Syn, USP14^+/−^ + AAV-GFP, and USP14^+/−^ + AAV-*h*α-Syn mice, respectively; *n* = 6, 7, 6, and 6 for female AAV-GFP, AAV-*h*α-Syn, USP14^+/−^ + AAV-GFP, and USP14^+/−^ + AAV-*h*α-Syn mice, respectively. Scale bar, 50 μm. Magnified images are shown on the right. Scale bar, 7 μm. (**e** and **f**) Representative blots and quantification showing the expressions of USP14, *h*α-Syn, and p-α-Syn (Ser129) in the striatum of male and female AAV-GFP, AAV-*h*α-Syn, USP14^+/−^ + AAV-GFP, and USP14^+/−^ + AAV-*h*α-Syn mice. *n* = 3 per group. (**g** and **h**) Representative ultrastructural images and quantitative analysis of synaptic vesicles in the SNpc of male and female AAV-GFP, AAV-*h*α-Syn, USP14^+/−^ + AAV-GFP, and USP14^+/−^ + AAV-*h*α-Syn mice. *n* = 6 per group. Scale bar, 500 nm. Results are expressed as mean ± SEM. ^**^p < 0.01, ^*^p < 0.05 vs. AAV-GFP; ^##^*p* < 0.01, ^#^*p* < 0.05 vs. AAV-*h*α-Syn. Statistical significance was determined using one-way ANOVA and Tukey’s test for *post hoc* comparisons

Next, we investigated the α-synuclein deposition. Exogenous human α-synuclein and phosphorylated α-synuclein deposition in the SN were increased in male USP14^+/−^ + AAV-*h*α-Syn mice, and the phosphorylated α-synuclein deposition was reduced in female USP14^+/−^ + AAV-*h*α-Syn mice, compared to AAV-*h*α-Syn mice (Fig. 6a and b). We further confirmed these findings using immunostaining assay of SNpc (Fig. 6c and d). In the striatum, the phosphorylated α-synuclein deposition was increased in male USP14^+/−^ + AAV-*h*α-Syn mice, while human α-synuclein deposition was decreased in female USP14^+/−^ + AAV-*h*α-Syn mice, compared with the respective AAV-*h*α-Syn mice (Fig. 6e and f). In addition, the number of synaptic vesicles was lower in male USP14^+/−^ + AAV-*h*α-Syn mice compared to AAV-*h*α-Syn mice, whereas no obvious changes were observed in female AAV-*h*α-Syn and USP14^+/−^ + AAV-*h*α-Syn mice (Fig. 6g and h). Overall, these results reveal that after AAV-*h*α-Syn injection, *Usp14* suppression augmented motor impairments and α-synuclein deposition in male mice, whereas these changes were attenuated in female mice.

### *Usp14* loss exerts different effects on microglial activation and pro-inflammatory response in male and female AAV-*h*α-Syn mice

**Fig. 7:**
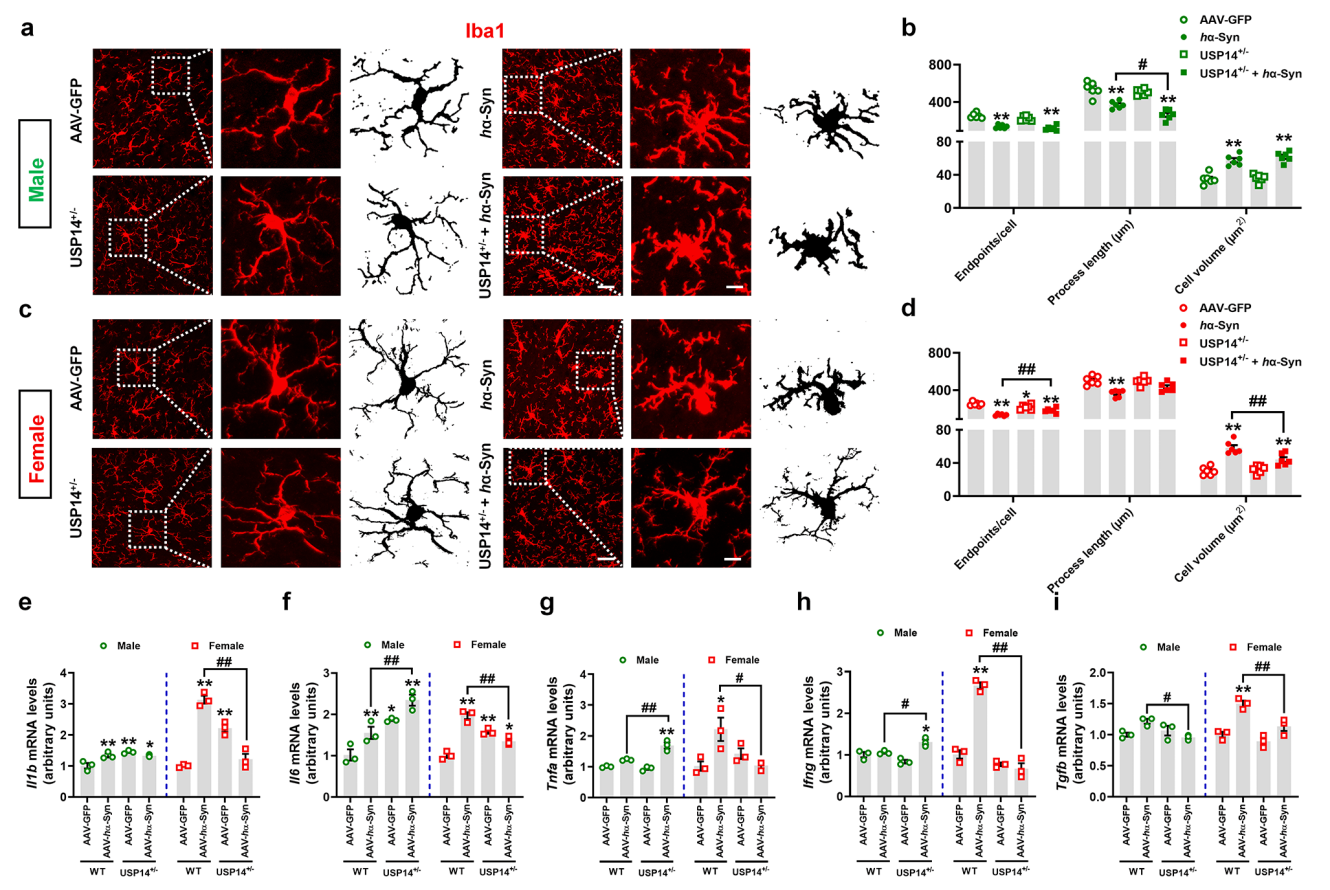
*Usp14* inactivation reduced the pro-inflammatory response in female AAV-*h*α-Syn-injected mice. (**a–d**) Immunostaining and quantification of Iba1-positive cells in the SNpc of male and female AAV-GFP, AAV-*h*α-Syn, USP14^+/−^ + AAV-GFP, and USP14^+/−^ + AAV-*h*α-Syn mice. *n* = 6 per group. Scale bar, 30 μm. Magnified images are shown in the middle. Scale bar, 12 μm. Skeletal images of Iba1-positive cells are shown on the right. (**e–i**) Nigral *Il1b*, *Il6*, *Tnfa*, *Ifng*, and *Tgfb* mRNA expressions were examined using qRT-PCR. *n* = 3 per group. Results are expressed as mean ± SEM. ^**^p < 0.01, ^*^p < 0.05 vs. AAV-GFP; ^##^*p* < 0.01, ^#^*p* < 0.05 vs. AAV-*h*α-Syn. Statistical significance was determined using one-way ANOVA and Tukey’s test for *post hoc* comparisons

Considering that S100A8/A9 may be related to the effects of α-synuclein burden in USP14^+/−^ mice, and S100A8/A9 mediates microglial activation in the brain [29, 30], we assessed the microglial function in SNpc. We found reduced microglial branch length in male USP14^+/−^ + AAV-*h*α-Syn mice compared to AAV-*h*α-Syn mice, whereas endpoints were increased and microglial body volumes were decreased in female USP14^+/−^ + AAV-*h*α-Syn mice compared to AAV-*h*α-Syn mice (Fig. 7a–d). These results suggest that microglia are activated in male AAV-*h*α-Syn + USP14^+/−^ mice and inhibited in female mice. In line with this, the mRNA expressions of pro-inflammatory cytokines (IL-6, TNF-α, and IFN-γ) were increased in the SN of male USP14^+/−^ + AAV-*h*α-Syn mice compared to AAV-*h*α-Syn mice. The mRNA expressions of IL-1β, IL-6, TNF-α, and IFN-γ were decreased in female USP14^+/−^ + AAV-*h*α-Syn mice compared to AAV-*h*α-Syn mice (Fig. 7e–h). The TGF-β mRNA expression was reduced in the SN of both male and female USP14^+/−^ + AAV-*h*α-Syn mice compared to AAV-*h*α-Syn mice (Fig. 7i). These data suggest that *Usp14* suppression in AAV-*h*α-Syn*-*injected mice induces microglial activation and pro-inflammatory response in male mice, whereas these changes are reduced in female mice.

### Differential effects of *Usp14* inactivation on male and female AAV-*h*α-Syn mice were mediated by S100A8/A9

**Fig. 8:**
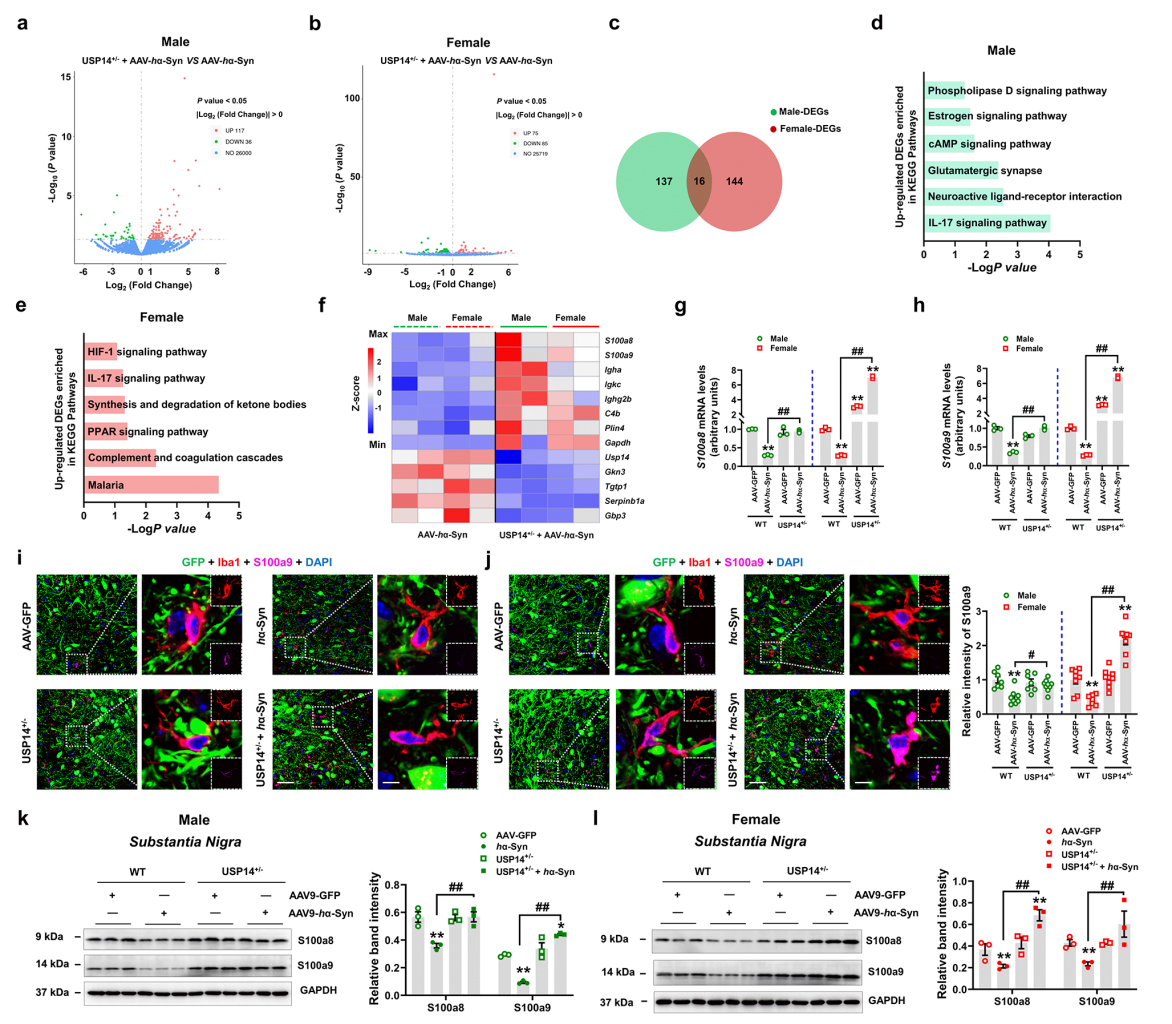
*Usp14* inactivation shows significant increase in S100a8/a9 expression in female AAV-*h* α-Syn-injected mice. (**a** and **b**) Volcano plot showing the DEGs between male and female USP14^+/−^ + AAV-*h*α-Syn mice and AAV-*h*α-Syn mice. (**c**) Venn diagram showing the overlapped genes between male and female USP14^+/−^ + AAV-*h*α-Syn vs. AAV-*h*α-Syn DEGs. (**d**) Kyoto Encyclopedia of Genes and Genomes (KEGG) pathways enriched by the upregulated DEGs between male USP14^+/−^ + AAV-*h*α-Syn mice and AAV-*h*α-Syn mice. (**e**) KEGG pathways enriched by the upregulated DEGs between female USP14^+/−^ + AAV-*h*α-Syn mice and AAV-*h*α-Syn mice. (**f**) Hierarchical clustering of common upregulated and downregulated DEGs between male and female USP14^+/−^ + AAV-*h*α-Syn vs. AAV-*h*α-Syn mice. (**g** and **h**) Nigral *S100a8* and *S100a9* mRNA expressions were detected using qRT-PCR. *n* = 3 per group. (**i** and **j**) Immunostaining and quantification of the S100a9 intensity in Iba1-positive cells in the SNpc of male and female AAV-GFP, AAV-*h*α-Syn, USP14^+/−^ + AAV-GFP, and USP14^+/−^ + AAV-*h*α-Syn mice. *n* = 7, 8, 7, and 9 for male AAV-GFP, AAV-*h*α-Syn, USP14^+/−^ + AAV-GFP, and USP14^+/−^ + AAV-*h*α-Syn mice, respectively; *n* = 8, 7, 9, and 8 for female AAV-GFP, AAV-*h*α-Syn, USP14^+/−^ + AAV-GFP, and USP14^+/−^ + AAV-*h*α-Syn mice, respectively. Scale bar, 50 μm. Magnified images are shown on the right. Scale bar, 5 μm. Results are expressed as mean ± SEM. ^**^p < 0.01 vs. AAV-GFP; ^##^*p* < 0.01, ^#^*p* < 0.05 vs. AAV-*h*α-Syn. Statistical significance was determined using one-way ANOVA and Tukey’s test for *post hoc* comparisons

Considering that the S100A8/A9 expression was increased in the SN of female USP14^+/−^; A53T-Tg mice, we further explored this finding in AAV-*h*α-Syn mice. The DEGs between male and female AAV-*h*α-Syn and USP14^+/−^ + AAV-*h*α-Syn mice are presented in Fig. 8a and b and Supplementary Fig. 2a and b. There were 16 DEGs shared by male and female mice (Fig. 8c). Kyoto Encyclopedia of Genes and Genomes analysis demonstrated that the pathways enriched by upregulated DEGs in male mice were mainly focused on synaptic function (such as glutamatergic synapse) and inflammatory response, whereas in female mice, they were mainly related to inflammatory signal modulation (such as complement and coagulation cascades and IL-17 signaling pathway) (Fig. 8d and e). We identified the common upregulated or downregulated DEGs between male and female AAV-*h*α-Syn and USP14^+/−^ + AAV-*h*α-Syn mice (Fig. 8f). Interestingly, we found increased *S100A8* and *S100A9* expressions in both male and female mice following AAV-*h*α-Syn injection in USP14^+/−^ mice (Fig. 8f). The expression upregulation between USP14^+/−^ + AAV-*h*α-Syn and AAV-*h*α-Syn was nearly 8-fold higher for female mice than male mice (Fig. 8g and h). Moreover, increased S100A9 expression was mainly found in the microglia of SNpc of female USP14^+/−^ + AAV-*h*α-Syn mice (Fig. 8i and j). Furthermore, phosphorylated α-synuclein expression was enhanced in CD68-positive microglia in the SNpc of female USP14^+/−^ + AAV-*h*α-Syn mice (Supplementary Fig. 3a and b). These results suggest that S100A8/A9 may stimulate microglia to engulf the pathological α-synuclein.

**Fig. 9:**
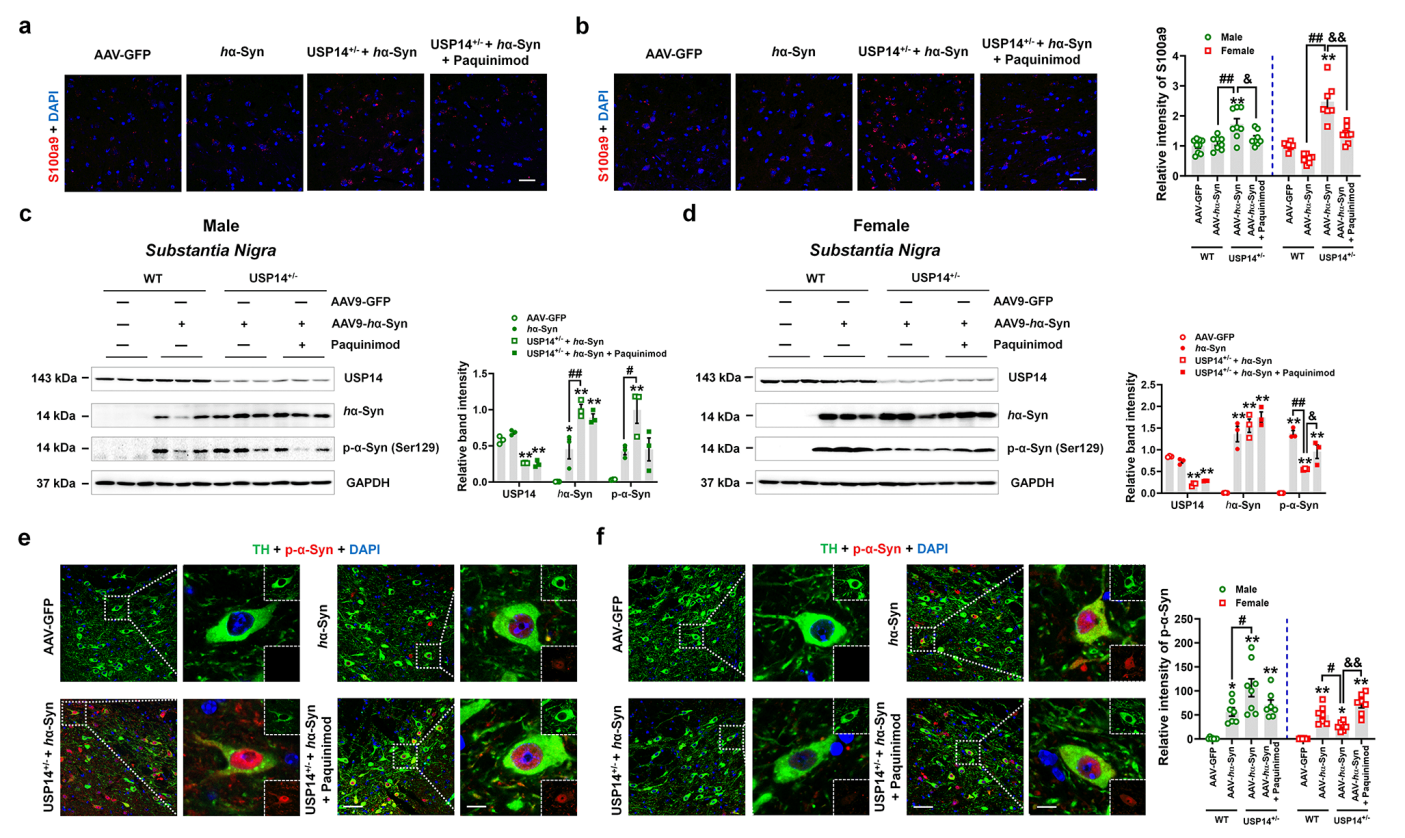
Pharmacological inhibition of S100A8/A9 reversed α-synuclein deposition in female USP14^+/−^+ AAV-*h* α-Syn-injected mice. (**a** and **b**) Immunostaining and quantification of S100A9 intensity in the SNpc of male and female AAV-GFP, AAV-*h*α-Syn, USP14^+/−^ + AAV-*h*α-Syn, and USP14^+/−^ + AAV-*h*α-Syn + paquinimod mice. *n* = 9, 8, 8, and 8 for male AAV-GFP, AAV-*h*α-Syn, USP14^+/−^ + AAV-*h*α-Syn, and USP14^+/−^ + AAV-*h*α-Syn + paquinimod mice, respectively; *n* = 7, 7, 7, and 8 for female AAV-GFP, AAV-*h*α-Syn, USP14^+/−^ + AAV-*h*α-Syn, and USP14^+/−^ + AAV-*h*α-Syn + paquinimod mice, respectively. Scale bar, 30 μm. (**c** and **d**) Representative blots and quantification showing the expressions of USP14, *h*α-Syn, and p-α-Syn (Ser129) in the SN of male and female AAV-GFP, AAV-*h*α-Syn, USP14^+/−^ + AAV-*h*α-Syn, and USP14^+/−^ + AAV-*h*α-Syn + paquinimod mice, respectively. *n* = 3 per group. (**e** and **f**) Immunostaining and quantification of the p-α-Syn intensity in the TH-positive cells in the SNpc of male and female AAV-GFP, AAV-*h*α-Syn, USP14^+/−^ + AAV-*h*α-Syn, and USP14^+/−^ + AAV-*h*α-Syn + paquinimod mice. *N* = 7, 7, 8, and 7 for male AAV-GFP, AAV-*h*α-Syn, USP14^+/−^ + AAV-*h*α-Syn, and USP14^+/−^ + AAV-*h*α-Syn + paquinimod mice, respectively; *n* = 6, 7, 7, and 7 for female AAV-GFP, AAV-*h*α-Syn, USP14^+/−^ + AAV-*h*α-Syn, and USP14^+/−^ + AAV-*h*α-Syn + paquinimod mice, respectively. Scale bar, 50 μm. Magnified images are shown on the right. Scale bar, 7 μm. Results are expressed as mean ± SEM. ^**^p < 0.01, ^*^p < 0.05 vs. AAV-GFP; ^##^*p* < 0.01, ^#^*p* < 0.05 vs. AAV-*h*α-Syn; ^&&^*p* < 0.01, ^&^*p* < 0.05 vs. USP14^+/−^ + AAV-*h*α-Syn. Statistical significance was determined using one-way ANOVA and Tukey’s test for *post hoc* comparisons

We evaluated the S100A8/A9 expression in response to the S100A8/A9 antagonist (Paquinimod). Paquinimod substantially reduced the S100A9 expression in the SNpc of male and female USP14^+/−^ + AAV-*h*α-Syn mice (Fig. 9a and b). There were no significant alterations in the expressions of human α-synuclein and phosphorylated α-synuclein in response to paquinimod in male USP14^+/−^ + AAV-*h*α-Syn mice (Fig. 9c). However, paquinimod reversed the decreased phosphorylated α-synuclein expression in female USP14^+/−^ + AAV-*h*α-Syn mice, whereas there were no significant effects on human α-synuclein expression (Fig. 9d). We further confirmed these results by staining for phosphorylated α-synuclein in dopaminergic neurons in the SNpc (Fig. 9e and f). These findings suggest that the effects of *Usp14* deficiency on the α-synuclein pathology in AAV-*h*α-Syn mice may be mediated by S100A8/A9.

### *Usp14* knockdown promotes autophagy and diminishes NF-κB activation in microglial BV2 cells

**Fig. 10:**
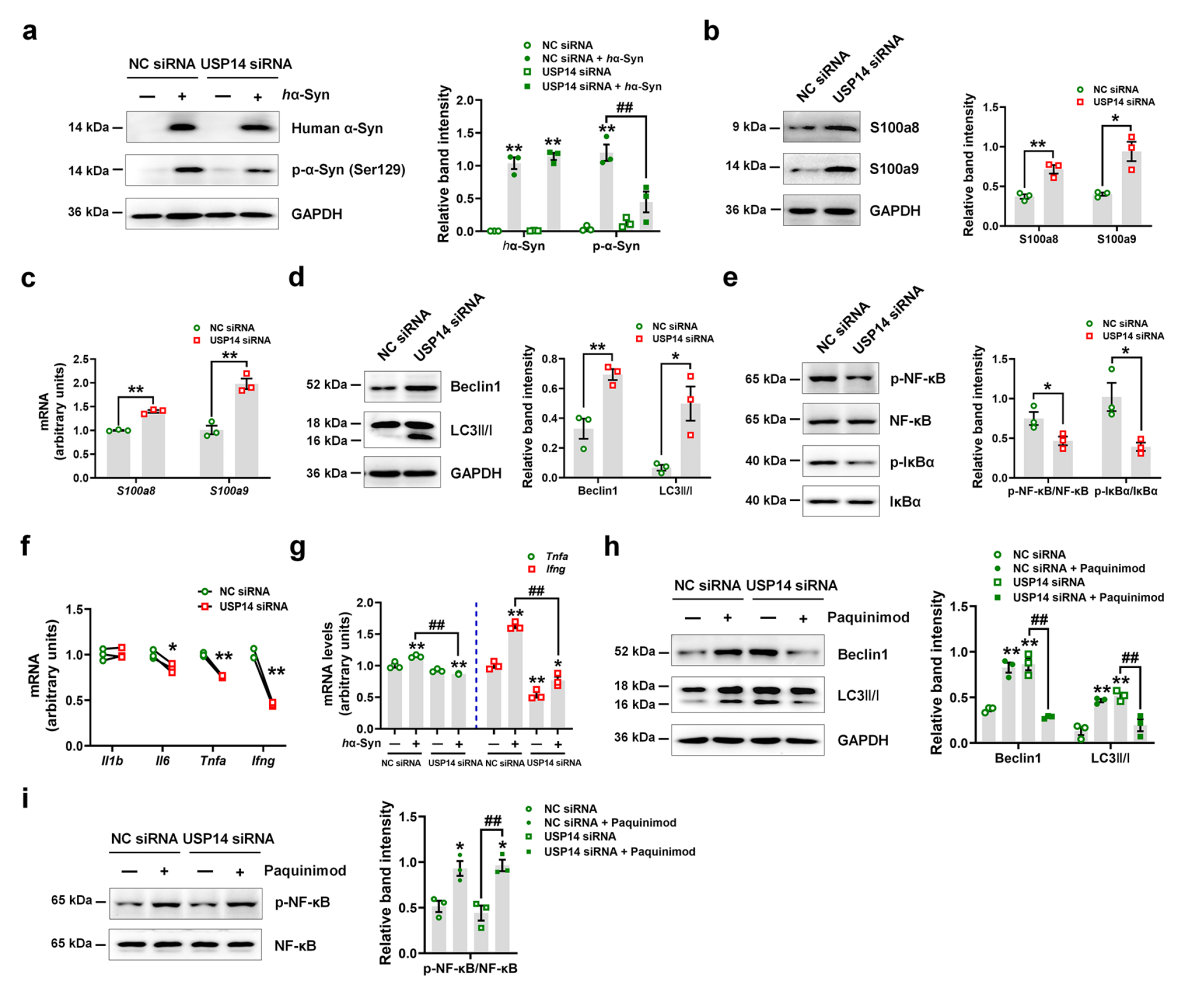
*Usp14* knockdown increases autophagy and inhibits NF-κB activation in microglial BV2 cells. (**a**) Representative blots and quantification showing the expression of *h*α-Syn and p-α-Syn (Ser129) after *Usp14* knockdown in *h*α-Syn fibril-treated BV2 cells. *n* = 3 per group. (**b** and **c**) Protein and mRNA expressions of S100a8 and S100a9 after *Usp14* knockdown in BV2 cells. *n* = 3 per group. (**d** and **e**) Representative blots and quantification showing the expressions of Beclin1, LC3 II/I, p-NF-κB, NF-κB, p-IκBα, and IκBα after *Usp14* knockdown in BV2 cells. *n* = 3 per group. (**f**) *Il1b*, *Il6*, *Tnfa*, and *Ifng* mRNA expression levels after *Usp14* knockdown in BV2 cells. *n* = 3 per group. (**g**) *Tnfa* and *Ifng* mRNA expression levels after *Usp14* knockdown in *h*α-Syn fibril-treated BV2 cells. *n* = 3 per group. (**h** and **i**) Representative blots and quantification showing the expressions of Beclin1, LC3 II/I, p-NF-κB, and NF-κB in Usp14 siRNA-treated BV2 cells with or without paquinimod. *n* = 3 per group. Results are expressed as mean ± SEM. ^**^p < 0.01, ^*^p < 0.05 vs. NC siRNA; ^##^*p* < 0.01 vs. NC siRNA + *h*α-Syn fibril or USP14 siRNA. Statistical significance was determined using Student’s *t*-test (b–f) and one-way ANOVA with Tukey’s test for *post hoc* comparisons (**a**, **g–i**)

Considering that USP14 negatively regulates autophagy and positively drives NF-κB activation [31, 32], consistent with our observation, we tested the hypothesis in microglial BV2 cells by interfering *Usp14* expression (Supplementary Fig. 4a and b; the second one was selected as the most efficient sequence). *Usp14* knockdown decreased the phosphorylated α-synuclein, but not human α-synuclein, expression in *h*α-Syn fibril-treated BV2 cells (Fig. 10a), which was consistent with our in vivo findings. Furthermore, *Usp14* knockdown increased the S100A8/A9 expressions at the protein and mRNA levels (Fig. 10b and c). *Usp14* knockdown increased the LC3 II/I ratio and Beclin 1 expression (Fig. 10d), suggesting that autophagy was increased. *Usp14* knockdown also suppressed NF-κB and IκBα phosphorylation and reduced the mRNA expressions of pro-inflammatory cytokines (IL-6, TNF-α, and IFN-γ) (Fig. 10e and f). On the other hand, *h*α-Syn fibril increased the mRNA expressions of pro-inflammatory cytokines (TNF-α and IFN-γ) and microglial receptors (CX3CR1 and CSF1R) (Supplementary Fig. 5A–C). Furthermore, *Usp14* knockdown decreased the mRNA expressions of TNF-α and IFN-γ (Fig. 10g). To explore whether the effects of *Usp14* knockdown are mediated by S100A8/A9, we administered paquinimod in vitro (Supplementary Fig. 6). Paquinimod attenuated the effects of *Usp14* knockdown on increasing the LC3II/I ratio and Beclin 1 expression, as well as inhibiting NF-κB activation (Fig. 10h and i). These findings suggest that the effects of *Usp14* knockdown on autophagy and NF-κB in BV2 cells are mediated by S100A8/A9.

There were no significant differences in CSF S100A8/A9 levels between controls and PD patients. Furthermore, we did not observe any correlation between CSF S100A8/A9 and α-synuclein (Supplementary Fig. 7a–h). In addition, we did not detect α-synuclein ubiquitination or an interaction between USP14 and α-synuclein in *h*α-Syn fibril-treated BV2 cells (Supplementary Fig. 8).

## Discussion

Previous studies have demonstrated that USP14 regulates neuronal survival, mitophagy, neuroinflammation, learning, memory, and neuromuscular junction structure and function [20–25, 33]. The effects of USP14 inhibition on aggregates, such as tau and huntingtin, in neurodegenerative diseases are controversial [19, 34, 35]. In this study, our findings regarding the effects of USP14 on α-synuclein in PD provides several insights.

First, CSF USP14 may be a promising indicator for PD. Recent accumulating evidence reveals that intact 20 S proteasomes in the circulatory system (c-proteasomes) and related plasma proteins represent pathophysiological conditions [36]. USP14 is associated with 19 S and 26 S proteasomes [14, 37], and it is conjugated with Ub, leading to 20 S proteasome gate opening [17], suggesting that USP14 may be involved in the production of c-proteasomes. In the present study, we detected decreased CSF USP14 expression in PD patients, particularly females. Furthermore, we found opposite relationships between CSF USP14 and α-synuclein expressions in male and female PD patients. Our results from USP14^+/−^ mice crossed with A53T-Tg or AAV-*h*α-Syn administration demonstrated that α-synuclein regulation by USP14 inactivation may be affected by gender, possibly due to the effects of hormones, such as estrogen. Conversely, we could not exclude the possibility of release of USP14 enzyme by damaged neural cells, such as neurons and microglia. Further studies are needed to examine the effects of circulating USP14.

Second, we evaluated the effects of *Usp14* inactivation on α-synuclein pathology. Proteasome activation was associated with USP14 inhibition, which clears the tau oligomer [19]. Furthermore, USP14 inactivation also induces autophagy by inhibiting K63 ubiquitination of Beclin 1, a key autophagy molecule [23, 38, 39]. In line with a previous study, we also found that *Usp14* knockdown increased Beclin 1 expression and autophagy, which may explain the observed clearance of α-synuclein deposition in female USP14^+/−^; A53T-Tg and USP14^+/−^ + AAV-*h*α-Syn mice. Mechanistically, we found that the effects of *Usp14* inactivation are mediated by S100A8/A9. S100A8 and S100A9 exist as heterodimers and are expressed in neutrophils and monocytes. S100A8/A9 activation in the brain can induce microglial activation [30, 40] and induce autophagy [41, 42]. Similar to our previous study and other studies, microglia can engulf the α-synuclein released by neurons by inducing microglial autophagy [43, 44]. We hypothesize that, under *Usp14* deficiency, S100A8/A9 promotes microglial phagocytosis of α-synuclein, thereby improving the behavioral abnormalities in female A53T-Tg and AAV-*h*α-Syn mice. Pharmacological inhibition of S100A8/A9 diminishes the effects of *Usp14* deficiency on α-synuclein clearance. Our study reveals a novel relationship between S100A8/A9 and DUBs in neurodegenerative diseases.

Finally, we found that *Usp14* inactivation reduced the pro-inflammatory response. Previously, USP14 was reported to mediate IκBα degradation and thus induce NF-κB activation [32]. USP14 inhibition suppressed the inflammatory reaction [45, 46]. As a potential downstream target of USP14 inhibition, S100A8/A9 usually triggers inflammation [47, 48] but also exerts anti-inflammatory effects by inducing IL-10 production, suppressing NF-κB activation, and reducing intracellular calcium signaling in immune cells [49–51]. In the present study, we found that *Usp14* knockdown suppressed NF-κB activation and reduced microglial activation and pro-inflammatory cytokine release in female A53T-Tg and AAV-*h*α-Syn mice. These neuroprotective effects were positively correlated with S100aA/A9 upregulation, whereas S100A8/A9 abolished the anti-inflammatory effects of *Usp14* knockdown. Our findings suggest that *Usp14* inactivation may promote microglial shift from the deleterious M1 phenotype to the neuroprotective M2 phenotype, leading to α-synuclein phagocytosis and an anti-inflammatory response. Next, we investigated how *Usp14* inactivation aggravates α-synuclein pathology and the pro-inflammatory response in male AAV-*h*α-Syn mice. *Usp14* inactivation increases S100A8/A9 deposition in male AAV-*h*α-Syn mice, although its level was much lower than that in female mice. We hypothesize that the higher S100A8/A9 expression can induce autophagy and exert anti-inflammatory effects. Furthermore, estrogen may play a role in determining the effects of *Usp14* deficiency.

Our results demonstrated a dual relationship between CSF USP14 and α-synuclein levels in PD patients. *Usp14* deficiency improved the behavioral abnormities and pathological α-synuclein deposition in female A53T-Tg and AAV-*h*α-Syn mice. Furthermore, *Usp14* inactivation attenuates the pro-inflammatory response in female AAV-*h*α-Syn mice. We also found that S100A8/A9 may be the downstream target of *Usp14* deficiency. Based on our findings, USP14 may represent a promising therapeutic target for PD.

## Materials and methods

### Patient characteristics

CSF samples were collected from 41 PD patients (mean age: 66.23 ± 7.42 years; 17 males and 24 females) and 41 healthy controls (mean age: 54.88 ± 13.69 years; 21 males and 20 females). The participants were recruited from the First Affiliated Hospital of Guangzhou Medical University. PD was diagnosed according to the UK Parkinson’s Disease Society Brain Bank clinical criteria [52]. The study protocol was approved by the Ethics Committee of the First Affiliated Hospital of Guangzhou Medical University. Written informed consent was obtained from all participants. Controls were matched with patients in terms of age and gender.

### Reagents

Anti-TH (F-11, sc-25,269) antibodies were purchased from Santa Cruz Biotechnology (Dallas, TX, USA). Anti-USP14 (#11,931), NF-κB p65 (#8242), Phospho-NF-κB p65 (Ser536) (#3033), S100A8 (#47,310), and S100A9 (#73,425) antibodies were purchased from Cell Signaling Technology (Danvers, MA, USA). Anti-human α-synuclein (ab138501), phospho-α-synuclein (Ser129) (ab51253), microtubule associated protein-1 light chain 3 (LC3) II/I (ab48394), CD68 (ab53444), phospho-IκBα (S36) (ab133462), and IκBα (ab32518) antibodies were purchased from Abcam (Cambridge, MA, USA). Anti-Beclin 1 (11306-1-AP) and GAPDH (60,004 − 1) antibodies were purchased from Proteintech Group (Rosemont, IL, USA). Anti-Iba1 (019 − 19,741) antibody was purchased from FUJIFILM Wako (Osaka, Japan). Paquinimod (HY-100,442) was purchased from MedChemExpress (Shanghai, China). DyLight 488 goat IgG (H + L) (70-GAM4882 and 70-GAR4882) and DyLight 649 goat IgG (H + L) (GAM649 and GAR649) antibodies were purchased from Multi Sciences (Hangzhou, China). Anti-rabbit IgG (H + L) (Alexa Fluor® 555 Conjugate, #4413S) and mouse IgG (H + L) (Alexa Fluor® 555 Conjugate, #4409S) antibodies were purchased from Cell Signaling Technology (Danvers, MA, USA). QuickBlock™ Blocking Buffer for Immunol Staining (P0260), Antifade Mounting Medium with DAPI (P0131), QuickBlock™ Blocking Buffer for Western Blot (P0252), and HRP-labeled Goat IgG (H + L) (anti-mouse, A0216; anti-rabbit, A0208) were purchased from Beyotime Biotechnology (Shanghai, China). α-synuclein enzyme-linked immunosorbent assay (ELISA) kits were purchased from Biolegend (San Diego, CA, USA). USP14 and S100a8/a9 ELISA kits were purchased from Enzyme-linked Biotechnology (Shanghai, China).

### ELISA

The CSF levels of α-synuclein, USP14, and S100A8/A9 were examined using ELISA kits according to the manufacturer’s protocols, as described previously [53]. Optical density values were determined using a Multiscan Spectrum (PerkinElmer, MA, USA) at 450 nm, and the results are expressed as pg/mL or ng/mL.

### BV2 cell culture and treatment

BV2 cells were procured from the American Type Culture Collection (Manassas, VA, ATCC) and were cultivated in DMEM (Gibco, C11995500BT) medium supplemented with 10% fetal bovine serum (Invitrogen, Carlsbad, CA, USA) at 37 °C under 5% CO2 in an incubator. Small interfering RNA (siRNA) targeting the *Usp14* sequence (siRNA1, 5′-GGAGAAGTTTGAAGGTGTA-3′; siRNA2, 5′- CAAGATGAATGGATCAAAT-3′; siRNA3, 5′- GAAGAGGTCACCAAAGGAA-3′) were custom-designed and synthesized by RioBio (Guangzhou, China). A negative-control siRNA was also provided by RioBio. The transfection procedure was similar to that described in our previous study [53]. Briefly, the siRNA (100 nM) was diluted and incubated with riboFECT CP Reagent (RioBio) for 15 min at room temperature (RT). Subsequently, the mixture was added to the culture medium and incubated with BV2 cells. To detect the effects of *Usp14* knockdown on α‑synuclein pathology, BV2 cells were transfected with USP14 siRNA for 48–72 h, and 5 µg/mL human α‑synuclein preformed fibrils was added during the final 24 h, as stated previously [44]. To examine the effects of S100A8/A9 inhibition after USP14 siRNA transfection, BV2 cells were treated with 50 µM paquinimod for the final 24 h. Then, the RNA and protein extracted from cellular samples were collected for RT-qPCR and Western blot analysis.

### Animals

Heterozygous USP14 knockout (USP14^+/−^) mice with a C57BL/6J genetic background generously provided by professor Jinbao Liu from Guangzhou Medical University. USP14^+/−^ mice were crossed with wild-type (WT) C57BL/6J mice, and the genotypes of the offspring were identified using PCR (primers: F 5’-TCCCAAAATAAATGTAAAGTCAAT and R 5’- AAGGTGGGTAGAGAAAATAAAAGT). hSNCA*A53T-Tg mice (also known as A53T α-Syn mice) were obtained from the Shanghai Model Organisms Center, Inc. (Shanghai, China). Crossbreeding between USP14^+/−^ and A53T transgenic mice resulted in four genetic combinations: WT, A53T-Tg, USP14^+/−^, and USP14^+/−^; A53T-Tg mice, which were further categorized in male and female subgroups. Adult (8-week-old) male and female C57BL/6J mice used for AAV-*h*α-Syn injection were procured from SPF Biotechnology Co., Ltd. (Beijing, China). The distribution of mice in each group was random and blinded, with age and sex being matched. Mice were housed in groups of 4–5 per cage under controlled conditions: a 12/12-h light/dark cycle, temperature of 22 ± 1 °C, and relative humidity of 60 ± 5%, with ad libitum access to food and water. The animal experiments were conducted in accordance with the National Institutes of Health Guide for the Care and Use of Laboratory Animals (NIH Publications No. 8023, revised 1978) and were approved by the Institutional Animal Care and Use Committee of Guangzhou Medical University.

### AAV‑*h*α‑Syn virus injection and drug treatment

Male and female WT and USP14^+/−^ mice were stereotactically injected with AAV‑*h*α‑Syn virus in bilateral SNpc (Bregma: AP, − 3.0 mm; ML, ± 1.3 mm; DV, − 4.7 mm) as reported previously [44]. Behavioral tests were performed after 8 weeks. To investigate the effects of S100A8/A9 inhibition on α-synuclein pathology, paquinimod (10 mg/kg) was administered intragastrically in AAV‑*h*α‑Syn-injected mice every other day for 1 month.

### Behavioral tests

#### OFT

OFT was performed similar to our previous study [44]. In brief, mice were placed within a 40 × 40 × 40-cm rectangular plastic enclosure, segmented into peripheral and central zones. Mice were allowed to explore freely for 15 min, and the movement speed, total distance traveled, and time spent in the central zone were recorded using a video tracking system (EthoVisione XT software, Beijing, China).

#### Grasping test

Grasping test was performed as described previously [44]. A horizontal stainless-steel wire with a diameter of 1 mm was suspended 30 cm above the ground. Mice were carefully placed so that their forepaws gripped the wire, and their grasping ability was observed for 10 s. Scores were assigned depending on whether the mice successfully grasped with both hind legs (3 points) or one hind leg (2 points), failed to grasp with either hind leg (1 point), or fell (0 points). Mice underwent three trials, and the average score was recorded.

#### Pole climbing test

Pole climbing test was performed as described previously [44]. Prior to the experimental procedure, mice were placed in a behavioral room for acclimatization for 30 min. The test involved a metal pole, with a diameter of 9 mm and length of 75 cm, wrapped in bandage gauze to enhance grip. Mice were gently positioned 7.5 cm away from the top of the pole. The time taken for each mouse to descend to the bottom was carefully recorded, with a maximum duration limit of 60 s for each trial.

#### Rotarod test

Rotarod test was performed similar to a previous study [44]. Before the test, mice underwent a 3-day training on a Rotarod apparatus (Ugo Basile SRL, Gemonio, VA, Italy) at a constant speed of 10 rpm. On day 4, mice were placed on the Rotarod, and the speed was gradually increased from 4 rpm to 40 rpm within 5 min. The duration spent by each mouse on the rotating rod before falling was accurately recorded. Trials were terminated if a mouse fell off the rod or clung to the apparatus for two full rotations without walking or running. Each mouse was subjected to three trials, and the mean duration was recorded.

#### Western blot

Brain tissues and cultured cells were lysed using a RIPA buffer containing 1 mM PMSF (Beyotime, Shanghai, China). The lysates were homogenized using ultrasonication and then centrifuged to collect the supernatant. Protein concentrations in the supernatant were quantified using the BCA Protein Quantification Kit (Beyotime, Shanghai, China). The lysates were separated via SDS-PAGE and subsequently transferred onto PVDF membranes. The membranes were blocked at RT for 30 min, followed by incubation with primary antibodies overnight at 4 °C and HRP-conjugated secondary antibodies for 1 h at RT. Protein bands were visualized using an enhanced chemiluminescence system with a luminol reagent (Beyotime, Shanghai, China). The chemiluminescent signals were captured using the GeneGnome XRQ Chemiluminescence system (Gene Company, Hong Kong, China) and measured using ImageJ software.

#### Quantitative polymerase chain reaction (qPCR)

The qPCR was performed as described previously [54]. Total RNA was extracted from cultured cells and SN tissues using TRIzol reagent (Invitrogen, San Diego, CA, USA). cDNA synthesis was performed using the cDNA Reverse Transcription Kit (Takara, Otsu, Japan). The qPCR was conducted using the Applied Biosystems 7500 Real-Time PCR System (Thermo Fisher Scientific, USA) with the following thermal cycling parameters: initial denaturation at 95 °C for 5 s, annealing at 55 °C for 30 s, and extension at 72 °C for 30 s. This cycle was repeated for 40 cycles to amplify the target sequences. TB Green Premix Ex Taq (Takara, Shiga, Japan) was used for the qPCR reactions, ensuring high sensitivity and specificity. Data were derived from three independent experiments, each with triplicate samples. The 2^−ΔΔCT^ method was used for data analysis, with *Gapdh* serving as the reference gene for normalization. Supplementary Table 1 presents the detailed information regarding the primers use.

#### Immunohistochemistry and immunofluorescence assays

The mouse brain was fixed in 4% paraformaldehyde and dehydrated in 20–30% sucrose buffer for 3 days. The brains were embedded in OCT (Solarbio, Beijing, China) and then coronally sectioned at a thickness of 30 μm using a Leica cryostat. For immunohistochemical staining, UltraSensitive™ SP (mouse/rabbit) IHC Kit (MXB KIT-9720, Fuzhou, China) was employed, involving DAB staining and subsequent dehydration and clearing. Imaging was conducted by scanning the slices using an optical microscope (CS2, Leica Microsystems, Germany). For immunofluorescence experiments, brain slices or fixed-cultured cells were blocked to prevent non-specific binding and then incubated overnight at 4 °C with primary antibodies, followed by incubation with fluorescently labeled secondary antibodies at RT for 1 h. The nuclei were counterstained with DAPI for visualization. The slides were imaged using a laser scanning confocal microscope (SP8, Leica Microsystems, Germany). The fluorescence intensity in these images was quantified using ImageJ software.

#### Transmission electron microscopy (TEM)

The ultrastructural morphology of synaptic vesicles in the SN tissue was analyzed using TEM, as stated previously [55]. Following euthanasia of mice, the SN was promptly extracted and immersed in the TEM fixative at RT away from light for 2 h. Subsequently, the specimens were preserved at 4 °C. The samples were washed three times with 0.1 M phosphate buffer (pH 7.4), sequentially dehydrated in an ascending ethanol gradient, incubated in 100% acetone for penetration, and embedded in epoxy resin (812 embedding agent) for final embedding. Ultra-thin Sects. (60–80 nm) were obtained using a Leica ultramicrotome and stained with uranyl acetate and lead citrate. Finally, images of synaptic vesicles were captured and subjected to further analysis using a TEM (HT7700, Hitachi, Tokyo, Japan).

#### RNA-seq

RNA-seq was performed as described previously [54]. In brief, RNA was extracted from SN using TRIzol (Invitrogen, Carlsbad, CA, USA). Subsequently, cDNA libraries were generated using the NEBNext® Ultra™ RNA Library Prep Kit for Illumina®, and these libraries were sequenced using a HiSeq 2500 instrument platform (San Diego, CA, USA). Novogene Inc. (Beijing, China) performed the sequencing process. Read counts were determined using HTSeq v0.6.0, and the fragments per kilobase of transcript-per-million mapped reads for each gene were calculated, taking into account the gene length and read counts mapped to it. DEGs were identified using predefined thresholds of fold change > 1, false discovery rate < 0.1, and adjusted *p* < 0.05. The pathways of DEGs were analyzed using the R package (v 3.5.1).

### Statistical analysis

Data are presented as mean ± standard error of the mean (SEM). Sample sizes were chosen based on the means and variation of preliminary data to achieve at least 80% power and allow for a 5% type I error. All calculations for sample sizes were done using an online sample size calculator (https://clincalc.com/stats/samplesize.aspx). Data were analyzed using Student’s *t*-test, one-way analysis of variance followed by Tukey’s *post hoc* test, as appropriate. Differences with a *p*-value < 0.05 were considered statistically significant. Statistical analyses were performed using GraphPad Prism 9.0 (GraphPad Software, La Jolla, CA, USA). *P*-values are represented as ^*^p < 0.05 and ^**^p < 0.01.

### Electronic supplementary material

Below is the link to the electronic supplementary material.

Supplementary Material 1

## Data Availability

All data supporting the current study are provided in the source data file. All additional information is available from the corresponding author upon request.
