# Supplementary material for: Usp14 deficiency removes α-synuclein by regulating S100A8/A9 in Parkinson’s disease
Source: Cell Mol Life Sci. 2024 May 23;81(1):232. doi: 10.1007/s00018-024-05246-8 (PMC11116365; doi:10.1007/s00018-024-05246-8)
Supplement: Supplementary file 1 — Supplementary Material 1 [file 18_2024_5246_MOESM1_ESM.docx]

**Supplementary Figures**





**Supplementary Figure 1. PCA plot for male and female A53T-Tg and USP14^+/-^; A53T-Tg mice.**





**Supplementary Figure 2. PCA plot for male and female AAV-*h*α-Syn and USP14^+/-^ + AAV-*h*α-Syn.**





**Supplementary Figure 3. Expression of p-α-Syn in CD68-positive cells.** (a and b) Immunostaining and quantification of the intensity of p-α-Syn in the CD686-positive cells in the SNpc of male and female AAV-GFP, AAV-*h*α-Syn, USP14^+/-^ + AAV-GFP, and USP14^+/-^ + AAV-*h*α-Syn mice. n = 6, 8, 8, 8 for male AAV-GFP, AAV-*h*α-Syn, USP14^+/-^ + AAV-GFP, and USP14^+/-^ + AAV-*h*α-Syn mice; n = 6, 9, 9, 10 for female AAV-GFP, AAV-*h*α-Syn, USP14^+/-^ + AAV-GFP, and USP14^+/-^ + AAV-*h*α-Syn mice. Scale bar, 25 μm. Magnified images were shown in the right. Scale bar, 4 μm. Results are expressed as mean ± SEM. ^**^*p* < 0.01 versus AAV-GFP; ^##^*p* < 0.01 versus AAV-*h*α-Syn. Statistical significance was determined using one-way ANOVA and Tukey’s test for *post hoc* comparisons.





**Supplementary Figure 4. Knockdown efficiency of USP14 siRNA in BV2 cells.** (a) Representative blots and quantification showing the expression of USP14 in the different siRNAs-treated BV2 cells. n = 3 per group. (b) mRNA expression of USP14 in the different siRNAs-treated BV2 cells. n = 3 per group. Results are expressed as mean ± SEM. ^**^*p* < 0.01 versus NC siRNA. Statistical significance was determined using one-way ANOVA and Tukey’s test for *post hoc* comparisons.





**Supplementary Figure 5. Effects of *h*α-Syn fibril on the inflammatory cytokines.** (a) Representative blots and quantification showing human α-synuclein expression in *h*α-Syn fibril-treated BV2 cells. n = 3 per group. (b and c) *Il1b*, *Il6*, *Tnfa*, *Ifng*, *Tgfb*, *Tmem119*, *Cx3cr1*, *Csf1r*, and *P2ry12* mRNA expressions were examined by qRT-PCR. n = 3 per group. Results are expressed as mean ± SEM. ^**^*p* < 0.01, ^*^*p* < 0.05 versus PBS. Statistical significance was determined using Student’s *t*-test.





**Supplementary Figure 6. Effect of Paquinimod on S100a8/a9 expression.** *S100a8* and *S100a9* mRNA expressions upon different concentrations of Paquinimod treatment in BV2 cells. n = 3 per group. Results are expressed as mean ± SEM. ^**^*p* < 0.01, ^*^*p* < 0.05 versus Ctrl. Statistical significance was determined using one-way ANOVA and Tukey’s test for *post hoc* comparisons.





**Supplementary Figure 7. The levels of S100a8/a9 in the CSF of male and female PD patients.** (a and b) The concentrations of S100a8/a9 in the CSF of male and female Control and PD patients were assessed by ELISA kits. n = 41 in Control group and n = 41 in PD group. n = 21 for male and n = 20 for female in Control group, n = 17 for male and n = 24 for female in PD group. (c-h) The correlation between S100a8/a9 and α-synuclein in Control and PD groups were performed by Pearson correlation analysis. Results are expressed as mean ± SEM. Statistical significance was determined using Student’s *t*-test.





**Supplementary Figure 8. The interaction between USP14 and α-synuclein.** Co-IP assay showing the representative blots of USP14, Ub and α-synuclein in *h*α-Syn fibril-treated BV2 cells, and α-synuclein was used as the capture antibody. Note the non-specific band in the Ub blot.

**Supplementary Table 1. Primer sequence used for qRT-PCR.**

| Mice genes | Primer sequence (5’-3’) |
| --- | --- |
| *S100a8* | F: GGAGTTCCTTGCGATGGTGAT  R: TCCTTGTGGCTGTCTTTGTGAG |
| *S100a9* | F: AGATGGCCAACAAAGCACCT  R: TAAAGGTTGCCAACTGTGCT |
| *Usp14* | F: CGGCTACCTGCTTACTTAACTA  R: TTAAGAACTTTGGCGTTCACAG |
| *Il1b* | F: AATGCCACCTTTTGACAGTGAT  R: TGCTGCGAGATTTGAAGCTG |
| *Il6* | F: AGGATACCACTCCCAACAGACC  R: AAGTGCATCATCGTTCATACA |
| *Tnfa* | F: CACGTCGTAGCAAACCACC  R: TGAGATCCATGCCGTTGGC |
| *Ifng* | F: TGGCAGGAGATGTCTACACT  R: GAAGCACCAGGTGTCAAGTC |
| *Tgfb* | F: ATTCCTGGCGTTACCTTGG  R: AGCCCTGTATTCCGTCTCCT |
| *Csf1r* | F: CCTCAAACGTGGAGACACCAA  R: CGTGTGCCAACATCATTGCT |
| *Cx3cr1* | F: CAACCCCTTTATCTACGCCTT  R: GACCCATCTCCCTCGCTTG |
| *Tmem119* | F: CTGACATTCTGGCTGCTACC  R: CACCCTTCACAGGCTTTGCTC |
| *P2ry12* | F: TTTGCTGGGCTCATCACGAAC  R: ACTGAAGTAACTTGGCACACC |
| *Gapdh* | F: ACGGGAAGCTCACTGGCATGGCCTT  R: CATGAGGTCCACCACCCTGTTGCTG |
